# Photo-ID and telemetry highlight a global whale shark hotspot in Palawan, Philippines

**DOI:** 10.1038/s41598-019-53718-w

**Published:** 2019-11-20

**Authors:** Gonzalo Araujo, Ariana Agustines, Brian Tracey, Sally Snow, Jessica Labaja, Alessandro Ponzo

**Affiliations:** Large Marine Vertebrates Research Institute Philippines, Cagulada Compound, Jagna, 6308 Bohol Philippines

**Keywords:** Conservation biology, Animal migration

## Abstract

The Philippines is home to the second largest known population of whale sharks in the world. The species is listed as endangered due to continued population declines in the Indo-Pacific. Knowledge about the connectivity within Southeast Asia remains poor, and thus international management is difficult. Here, we employed pop-up archival tags, data mining and dedicated effort to understand an aggregation of whale sharks at Honda Bay, Palawan, Philippines, and its role in the species' conservation. Between Apr and Oct 2018, we conducted 159 surveys identifying 117 individual whale sharks through their unique spot patterns (96.5% male, mean 4.5 m). A further 66 individual whale sharks were identified from local operators, and data mined on social media platforms. The satellite telemetry data showed that the whale sharks moved broadly, with one individual moving to Sabah, Malaysia, before returning to the site <1 year later. Similarly, another tagged whale shark returned to the site at a similar periodicity after reaching the Malay-Filipino border. One individual whale shark first identified in East Kalimantan, Indonesia by a citizen scientist was resighted in Honda Bay ~3.5 years later. Honda Bay is a globally important site for the endangered whale shark with connectivity to two neighbouring countries, highlighting the need for international cooperation to manage the species.

## Introduction

The whale shark *Rhincodon typus* Smith 1828 is the world’s largest extant fish, capable of reaching a maximum size of 19.6 m in length^1^. It inhabits tropical and warm temperate waters^2^, and aggregates to feed in numbers of up to a few hundred^3^. These predictable aggregations occur at various sites across the globe to prey on sergestid shrimps (e.g. Mafia Island, Tanzania^4^; Bahia de Los Angeles, Mexico^5^), fish spawn (e.g. Belize^6^; Qatar^7^; Caribbean Mexico^8^), coral spawn (e.g. Ningaloo Reef^9^), or on provisioned food (e.g. Oslob, Philippines^10^; Cenderawasih Bay, Indonesia^11^) amongst others.

The whale shark is listed as ‘Endangered’ under the IUCN Red list of Threatened Species^12^ due to declining population numbers, particularly in the Indo-Pacific region. Though the species is protected nationally in countries that used to operate targeted fisheries (e.g. Taiwan, India, Philippines), concerns remain from ongoing fisheries in the south of China where over 1,000 animals are reportedly landed yearly in the Hainan province alone^13^, and a Wild Life Risk report where a single shark processing factory in the Zhejiang province processed up to 600 whale sharks per year^14^. These numbers are substantial considering, for example, that in 22 years (1992–2014) of photographic identification at Ningaloo Reef, Western Australia, a total of 1,082 individuals were identified^15^. The species was listed into Appendix I of the Convention on Migratory Species in 2017, highlighting the need for international efforts to enhance their conservation, given crucial gaps in our knowledge of their life history and the decline in numbers observed across multiple sites (CMS/UNEP/CoP12, 2017).

Documented whale shark aggregations are normally dominated by juvenile males ranging from 4 to 8 m in length^2^, with the exception of Darwin, Galapagos Islands^16^, and Baja California^17^, where adult females are frequently sighted. In the Arabian Gulf, a high proportion of adult males and females were reported at an offshore aggregation in Qatar^7^, and at St Helena Island in the South Atlantic^18^. Similarly, whale sharks visiting Donsol in the Philippines had a high proportion of mature males (53%)^19^. Assuming an expected 1:1 birth ratio as observed in Taiwan^20^, no data is available as to the whereabouts of juvenile females, with the exception of Saudi Arabia’s Red Sea where the first known 1:1 juvenile aggregation was reported^21^. The occurrence of neonates is negligible with very few encounters across the world documented to date, those of which come mostly from fisheries interactions^22,23^. Shifts in ontogenetic habitat use are important to help identify critical habitats for this endangered species, particularly if international boundaries are crossed.

The whale shark has a unique spot pattern that allows for the identification of uniquely marked individuals through photographic identification (photo-ID), and subsequently for mark-recapture studies^24^. Photo-ID is a cost-effective minimally invasive technique used to describe population dynamics^25^. These methods have now been employed to understand population dynamics at most whale shark sites across the globe (e.g. Ningaloo Reef^9^; Qatar^7^). Specifically, modified maximum likelihood methods can be employed to elucidate their lagged identification rate (LIR), defined as the probability of recapturing an animal after a certain time lag^26^, to understand their local ecology. This approach uses the identification data itself, including from several sources^27^, to estimate various population parameters such as population size, residency, mortality, etc. The surface-dwelling and slow-moving nature of the whale shark, coupled with its unique individual spot patterns^24^, makes it an ideal candidate species for citizen science projects^25,28^. Citizen science, by which the general public is enlisted to participate in scientific projects, is a powerful tool that can help monitor ecological and environmental factors, respond to crises, or inform management actions on a local, regional or global scale^29–33^. In marine megafauna species, citizen science has been used to understand abundance and demographics, distribution, and threats amongst others (e.g. reef sharks^34^, wobbegong shark^35^; humpback whales^36^; green turtles^37^). In whale sharks specifically, citizen science and data mining contributions have aided the understanding of their habitat use and connectivity across different countries, demographics and life-history traits^15,27,38^. Data can also be extracted by mining historical social media posts (e.g. ©YouTube, ©Facebook), and thus also contribute to scientific projects^19,27,28^.

Telemetry can complement photo-ID and help understand habitat use and movements, as has been shown in whale sharks. In Madagascar, Diamant *et al*.^38^ satellite-tracked eight juvenile whale sharks to identify unknown foraging grounds, and photo-ID was used to understand periodicity at the site and connectivity to other regional sites where dedicated photo-ID programmes are active. Robinson *et al*.^39^ showed how whale sharks in the Arabian Gulf spent the majority of their time within the Gulf with annual returnees to the site, and strong site fidelity to the tagging site. Rohner *et al*.^40^ tracked whale sharks in the Mozambique coastline making regular international movements to South Africa, and Hearn *et al*.^41^ showed the long-distance movement of adult females from the Galapagos Islands. Telemetry can thus be employed to help our understanding of an endangered species, their habitat preference and use, local and afar movements, and any connectivity to other countries— essential data for their effective management.

The Bohol Sea, Philippines, was an active whale shark hunting ground until the late 1990s when the species was nationally protected (FAO 193, Department of Agriculture). Alava *et al*.^42^ reported *ca*. 500 whale sharks landed between 1993 and 1997 at just two localities, with a decrease in CPUE between these years. Following the ban on whale shark hunting, tourism endeavours started in the country, with Donsol, Sorsogon Province, leading the way^43^. The site quickly attracted up to 27,000 tourists per season^19^. Another ecotourism initiative started in Pintuyan, Southern Leyte, in 2006, with varying seasonal occurrence of whale sharks from December to June^44^. These tourism endeavours were masked by a different kind of whale shark tourism that emerged in Oslob, Cebu, where whale sharks are provisioned daily, year-round, and now receives >500,000 tourists a year [10, Oslob Tourist Logbook 2019]. Whale sharks are also seasonally (Mar-Jun) reported at Tubbataha Reefs Natural Park (TRNP) in the Sulu Sea and recent evidence suggest these sharks move broadly through the region^45^. Some evidence of whale sharks in Honda Bay, Palawan, exists through reports of sightings in September and October, as well as direct take, though nothing suggests this was an ongoing targeted fishery for the species^46^.

Here, we investigate the population dynamics of whale sharks in Honda Bay, Palawan, Philippines, and its relevance globally. We use pop-up archival tags to understand regional movements and habitat use, and data mining with dedicated photo-ID effort to estimate population size and residency through modified maximum likelihood methods. We discuss how these can inform conservation and management initiatives for this endangered species in the region.

## Results

Using dedicated effort and data mined from different sources, we identified a total of 183 individual whale sharks, of which 109 were male following clasper inspection and 4 were female, and 70 whose sex could not be determined. There was a considerable male bias for those sexed individuals (96.5% male; χ^2^ = 59.9, *P* < 0.001). The estimated total length (*T*_*L*_) of individually identified whale sharks was 4.46 ± 1.08 m (range 2.25–8.00 m). Only one male was considered mature based on clasper visual inspection, estimated at 8.00 m *T*_*L*_.

### Survey effort and photo-ID

**Figure 1:**
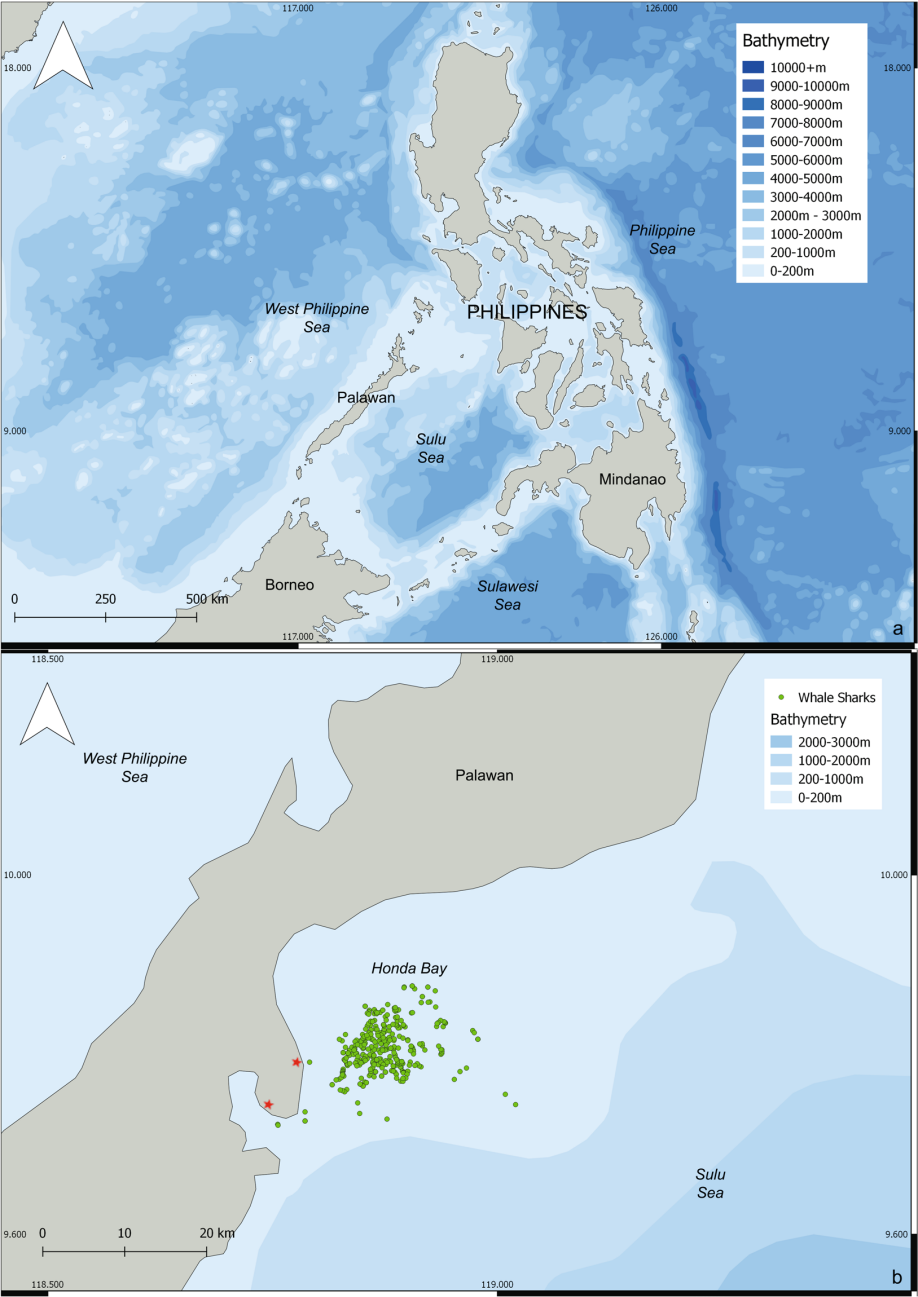
Map of the Philippines (**a**) and of Honda Bay in Palawan (**b**). The green dots represent whale shark encounters between Apr and Oct 2018. The red stars represent the survey start points.

We conducted a total of 159 surveys between Apr 26 and Oct 21, 2018, encountering at least one individual whale shark on 63% of surveys. Surveys conducted onboard tourist boats encountered at least one whale shark on 89% of instances while those from the pumpboat encountered at least one whale shark on 49% of the surveys. Pumpboat surveys lasted an average of 04 hr 58 min, covering an average of 45.3 km, whereas surveys onboard tourist boats lasted 05 hr 35 min covering 69.7 km. Overall, we had a total of 507 whale shark encounters leading to 419 photo identifications with 117 individual whale sharks, with a mean of 2.6 successful encounters (identified individual whale shark) per survey. All whale sharks were encountered within the southern half of Honda Bay (Fig. 1b).

### Data mining and other sources

In total, we extracted a total of 230 images yielding 106 unique IDs. Of these, 66 were unique left IDs assigned as newly identified individuals to the Philippines database on ‘Wildbook for Whale Sharks’ (Supplementary Table 1). A further 20 identification images were used from preliminary work by the Authors (May–Jun 2016, n = 10) and those collected during the whale shark tagging in Jul 2017 (n = 10). These 86 unique records were used to provide temporal information of whale sharks in Honda Bay.

### Movements from Photo-ID

One whale shark first reported at Tubbataha Reefs Natural Park (TRNP, ~160 km SE of Honda Bay) on Apr 15 2015 was resighted in Honda Bay on May 21 2016. Another whale shark first reported in TRNP in May 2003 (date unspecified) was resighted in Honda Bay on Oct 20 2008. Two whale sharks identified in Honda Bay were matched to Oslob, Cebu (~500 km E of Honda Bay). Individual P-745 was first identified in Oslob on Nov 24 2014 and resighted in Honda Bay on Apr 24 2016 by a citizen scientist. Individual P-730 was first identified in Oslob on Oct 06 2014 and resighted in Honda Bay on Oct 07 2018 whilst on survey.

More interestingly, individual ID-051, a 3 m male, was first identified in East Kalimantan, Indonesia on Dec 29 2013 by a citizen scientist who submitted the encounter to Wildbook for Whale Sharks, and was resighted in Honda Bay on Oct 19 2018 whilst on survey. On this occasion the shark was visually estimated to measure 4 m *T*_*L*_. This represents the first international whale shark match between the Philippines and Indonesia using photo-ID.

### Lagged identification rate and residency

**Figure 2:**
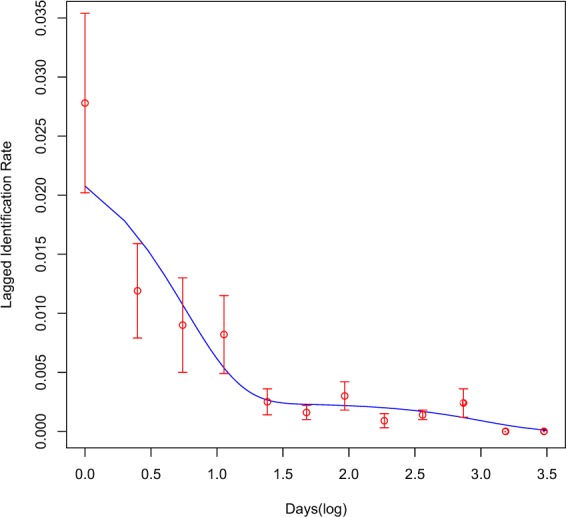
Estimated lagged identification rate for whale sharks at Honda Bay based on modified maximum likelihood methods adapted from Whitehead^26^.

Model H (Table 1) best fitted the empirical data through a combination of mortality, emigration, reimmigration and residency parameters. The LIR declined quickly following initial identification (Fig. 2) and continued to decline before rising after mean 92.9 days, then again after mean 362.0 days, and 738.3 days, and never quite reaching zero after 1,539.2 days. The model estimated a mean 41.1 ± 13.5 whale sharks at the study site at any one time, residing a mean 6.4 ± 2.9 days within the study area, whilst spending 58.2 ± 25.5 days outside. Mortality or permanent emigration rate was estimated at 0.00097 ± 0.00056.

**Table 1:** Model results for modified maximum likelihood methods using parameters to test for population closure, mortality or permanent emigration, reimmigration and residency as preset in program SOCPROG 2.7^52^.

Model Name	Parameters	ΔQAIC
A	Closed (1/a1 = N)	41.6
B	Closed (a1 = N)	1,129.2
C	Emigration/mortality (a1 = emigration rate; 1/a2 = N)	22.1
D	Closed: Emigration + reimmigration (a1 = emigration rate; a2/(a2 + a3) = proportion of population in study area at any time)	17.0
E	Emigration/mortality (a1 = N; a2 = Mean residence time)	22.1
F	Emigration + reimmigration + mortality	6.5
G	Emigration + reimmigration (a1 = N; a2 = Mean time in study area; a3 = Mean time out of study area)	4.4
**H**	**Emigration + reimmigration + mortality (a1 = N; a2 = mean time in study area; a3 = mean time out of study area; a4 = mortality rate)**	**0.0**

*N* = population size; QAIC: quasi-Akaike information criterion.

### Whale shark tracking

**Figure 3:**
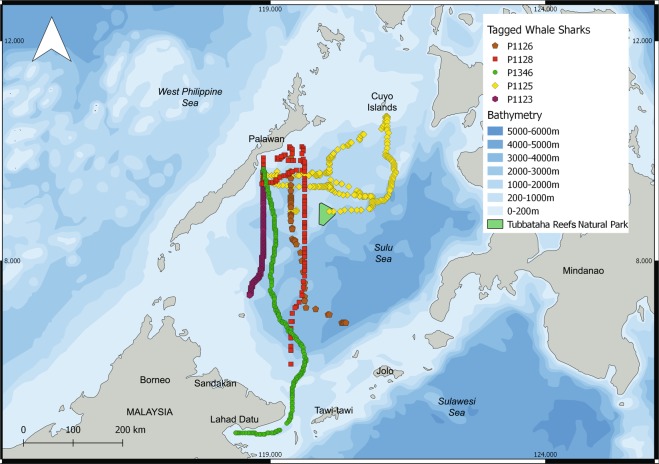
Estimated most probable track of five whale sharks tagged in Honda Bay, Palawan, Philippines in Jul 2017 using light locations. Note that only tags from whale sharks P-1126 and P-1125 transmitted sufficient light level data points to estimate short-term horizontal movement. An animation with confidence intervals is attached as Supplementary Video 1.

Only five of the ten miniPAT tags popped up and transmitted any data. The five sharks were tracked for a mean 136.6 ± 51.3 days (range 61–200 days). Of these 5, data transmission was very limited (summary in Table 2), and none were physically retrieved. Four of the 5 tracked individuals (P-1346, P-1128, P-1126, P-1123) moved south between deployment and pop-up date (Fig. 3). Given the limited data transmitted, and the spatial accuracy of these tags (~50 km), the movement presented here is an estimate and not absolute (an animation with 50%, 95% and 99% confidence intervals is presented as Supplementary Video 1). However, it is clear that these tracked animals moved south during the overall tracking period (Fig. 3). Individual P-1125 was tracked for a total of 200 days and transmitted more complete data packages of its locations. The animal appears to have first moved northeast towards Cuyo Islands in the northern Sulu Sea, before returning south via the Cagayancillo archipelago and TRNP, where the tagged popped-up southeast of Honda Bay (Fig. 3).

**Table 2:** Summary of whale sharks tagged in Honda Bay, Palawan, with pop-archival tags in July 2017.

Tag	Shark ID	Sex	Size (m)	Deployment date	Pop-up date	Tracked duration (d)	Distance (km)*	Max. depth (m)	Min. depth (m)	Max. temperature (°C)	Min. temperature (°C)	Light locations
39706	P-1123	M	4.0	20-Jul-17	26-Dec-17	159	315.9	381.5	0	n/a	n/a	42
39707	P-1126	M	3.5	21-Jul-17	20-Sep-17	61	395.4	516.0	0.5	n/a	n/a	123
39710	P-1346	M	2.5	19-Jul-17	08-Dec-17	142	558.9	448.5	1	30.6	10.7	16
39721	P-1125	M	4.0	21-Jul-17	06-Feb-18	200	111.0	953.0	0.5	n/a	n/a	279
39729	P-1128	M	4.5	23-Jul-17	21-Nov-17	121	389.7	1009.5	0.5	n/a	n/a	232
39701	P-1396	M	4.0	22-Jul-17	n/a	n/a	n/a	n/a	n/a	n/a	n/a	n/a
39720	P-1122	M	3.5	19-Jul-17	n/a	n/a	n/a	n/a	n/a	n/a	n/a	n/a
39741	P-1124	M	6.0	20-Jul-17	n/a	n/a	n/a	n/a	n/a	n/a	n/a	n/a
39742	n/a	n/a	5.0	22-Jul-17	n/a	n/a	n/a	n/a	n/a	n/a	n/a	n/a
39748	P-1127	M	2.5	22-Jul-18	n/a	n/a	n/a	n/a	n/a	n/a	n/a	n/a

*Distance (km) reflects the minimum straight line between deployment and pop-up location. Tags #39701, #39720, #39741, #39742 and #39748 failed to transmit any data.

At least three of the five tracked whale sharks returned to Honda Bay. Two from the animals that transmitted data (P-1125 and P-1128; Fig. 3), and P-1122 which was resighted by citizen scientists on the Oct 30 2017 still carrying a heavily fouled tag (Table 3). Individual P-1396 was resighted in Honda Bay during whale shark surveys on the Jul 7 2018 not carrying the tag and no obvious scarring to the naked eye.

**Table 3:** Whale sharks initially tagged in Honda Bay and resighted at the tagging site.

Shark ID	Resighting date	Resighting location	Time after tagging (d)	Remarks
P-1122	30-Oct-17	Honda Bay	103	Sighted by a citizen scientist; tag heavily fouled (see Supplementary Fig. 1)
P-1346	28-May-18	Honda Bay	313	No tag attached, only tether
P-1396	07-Jul-18	Honda Bay	350	No tag, no tether, no scar observed (see Supplementary Fig. 2)
P-1128	23-May-18	Honda Bay	304	No tag attached, only tether
P-1128	24-May-18	Honda Bay	305	No tag attached, only tether

At least one whale shark (P-1346) moved to Malaysian waters, to Lahad Datu Bay in north-eastern Sabah, Borneo (Fig. 3). Individual P-1128 appeared to have been following a similar path, but the tag popped up northeast of the Turtle Islands, near the Malay-Filipino border. Both P-1128 and P-1346 were resighted in Honda Bay on May 23 and 28 2018 respectively during whale shark surveys (Table 3), representing the first confirmed international return of whale sharks in Asia.

### Time-at-depth

**Figure 4:**
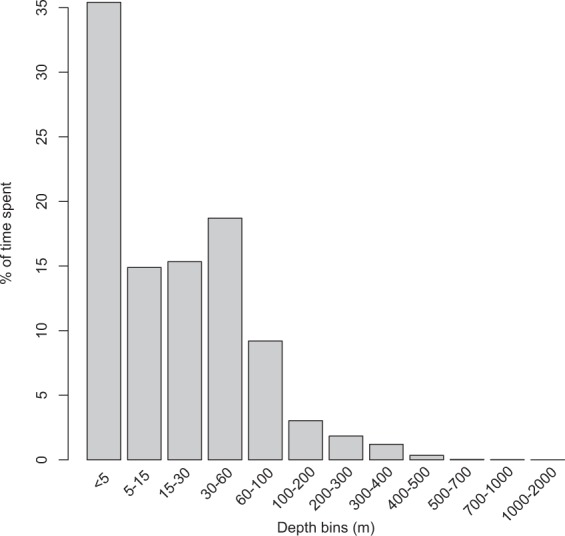
Time-at-depth of histograms received from 5 sharks, combined.

A total of 404 12-hr time at depth (TAD) histograms were received from all five tags. Sharks used all depth bins, including the deepest (1,000–2,000 m). Whale sharks spent the majority of their time in the epipelagic zone (96.6%), with the majority of this time spent at the top 5 m (35.4%), followed by the 30–60 m bin (18.7%), the 15–30 m bin (15.3%) and the 5–15 m (14.9%; Fig. 4). Overall sharks displayed some difference in their TAD patterns; however, differences in the number of histograms transmitted per tag differed greatly (range 13–180).

### Time-at-temperature

**Figure 5:**
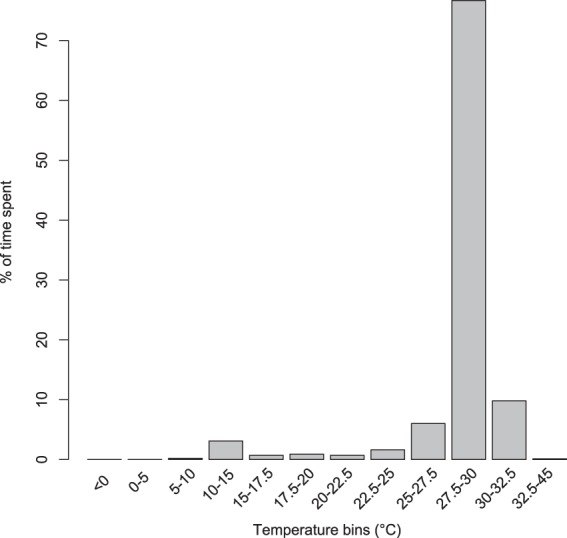
Time-at-temperature of histograms received from 5 sharks, combined.

A total of 411 12-hr time at temperature (TAT) histograms were transmitted from all five tags (Fig. 5). Sharks utilised all temperature bins between 5–10 °C and 32.5–45 °C. Sharks spent the majority of their time (76.7%) in the 27.5–30 °C bin, corresponding with the TAD of 0–60 m depth use. Overall, sharks spent only 5% of their time in waters <20 °C, with some time spent (3.3%) at temperatures <15 °C.

### Diving

To estimate the vertical velocity during descent, we selected dives that were characterised by a clear V-shape, and where sharks went from shallower water (<50 m) to >500 m deep. Given the gaps in the time-series data transmitted (intervals of 7.5–10 min), we present estimates that are indicative of the minimum vertical velocity of descent for sharks P-1125, P-1126 and P-1128. A total of 11 dives >500 m were recorded amongst these three sharks (range 516–1,009.5 m). The mean descent vertical velocity was estimated at 0.32 ± 0.29 ms^−1^ (range 0.004–0.988 ms^−1^). For individual P-1128, all dives >500 m (n = 7) took place between 04:40 pm and 07:40 am, whereas individual P-1126 only dive >500 m took place at 12:52 pm, and P-1125 dives were at 07:00 am, 03:30 pm and 10:00 pm.

A total of 189 deep dives (>200 m) were performed by sharks P-1125, P-1126 and P-1128 between Jul 22 2017 and Jan 18 2018. Two thirds (67.7%) of these dives were performed between 06:00 pm and 05:59 am, with the remainder of deep dives (32.3%) taking place between 06:00 am and 05:59 pm, daylight hours in the region. Most deep dives were transmitted from individual P-1125 (98), consistently performing a third (34.7%) of deep dives during between 06:00 am and 05:59 pm. Some periodicity was observed in the temporal deep diving of all sharks, with marked absences of deep dives (Supplementary Fig. 3).

## Discussion

Honda Bay, Palawan, is a globally important whale shark hotspot with 117 individuals identified in a single season through dedicated photo-ID, and a further 66 identified through data mined from social media platforms and other sources. This small juvenile, male-dominated aggregation appears to occur seasonally between Apr and Oct, with some individuals returning yearly to feed on small fishes and krill. Through photo-ID, individuals were matched to other sites in the Philippines. Combined with satellite telemetry, we report the first international return movement of whale sharks in Asia, with one individual moving to Lahad Datu in Malaysian Borneo through the Tawi-Tawi Kinabatangan strait, and returning to Honda Bay less than a year later. A second shark was also tracked to the edge of the Malay-Filipino border in the south Sulu Sea, and the animal returned to Honda Bay *ca*. a year later. Two other tags also moved to the southern Sulu Sea, and the fifth tag that transmitted travelled north before returning to the central Sulu Sea. We also report the first photo-ID match between the Philippines and Indonesia by that of a small male identified in East Kalimantan, Indonesian Borneo, in late 2013, and resighted in Honda Bay during our 2018 seasonal work.

Photo-ID was effectively employed to identify 183 individual whale sharks at Honda Bay, with a significant contribution (36%) from the general public as citizen scientists. The slow-swimming, and relatively benign nature of the whale shark makes it easy to photograph these animals underwater^28^, however, the quality of the photographs needs improving. The number of identification images usable from the number of encounters obtained through other data sources was relatively low (66 out of 230). An educational process to the tourists, perhaps as part of the interaction briefing, about the importance of collecting photo-ID data and how they can independently submit their encounters to Wildbook for Whale Sharks, would maximise data collection^15^. Not only can the data be used for mark-recapture models, but it can also provide insight into the size, sex and threats (e.g. propeller cuts, rope entanglement) individual whale sharks are exposed to.

Whale sharks visiting Honda Bay were mostly juveniles (mean 4.5 m total length), similar to that observed in Djibuti (3.7 m)^47^, Saudi Arabia (4.3 m)^21^, Bahia de la Paz, MX (4 m)^17^, Taiwan (4.6 m)^48^ and Christmas Island (4.6 m)^49^. They are however considerably smaller than whale sharks observed elsewhere in the Philippines as estimated visually (Cebu, 5.5 m^10^, Southern Leyte, 5.7 m^27^, Donsol, 6.5 m^19^), and at other Indo-Pacific aggregations like Mozambique (6.7 m)^50^, Qatar (6.9 m)^7^ or the Galapagos Islands (10.4 m)^41^. It appears Honda Bay might play a role in the developmental stage of these small juvenile whale sharks, particularly for males (96.5% of identified individuals). Only one adult male was observed, suggesting this is unlikely a mating ground for the species, but rather a targeted or opportunistic foraging ground.

Honda Bay is a globally important site for whale sharks, with maximum likelihood methods estimating a mean ~41 whale sharks at any one time within the survey area. Using similar methods, Araujo *et al*.^27^ estimated a mean ~16 individual whale sharks at Panaon Island in Southern Leyte, McCoy *et al*.^19^ a mean ~53 whale sharks at Donsol, Sorsogon, and daily, year-round effort at Oslob, Cebu, had a mean of 18.6 whale sharks weekly^51^. Similarly, Prebble *et al*.^52^ estimated ~35 whale sharks at Mafia Island, Tanzania, ~51 whale sharks at Mozambique and ~116 at an offshore aggregation in Qatar. Cochran *et al*.^21^ estimated ~21 individual whale sharks at a juvenile aggregation in Saudi Arabia (Red Sea) and Fox *et al*.^53^ estimated ~5 whale sharks at Utila, Honduras. The numbers observed in Honda Bay are comparatively significant and makes it the second largest aggregation in the Philippines, an important factor given the various laws governing the species nationally and the history of targeted hunting here^42^.

Whale sharks were encountered active feeding in small tuna boils, targeting the same prey the tunas were. We identified the main species as the Philippine anchovy *Encrasicholina oligobranchus*, although other species were probably present throughout the season. On occasion, the sharks were encountered feeding on krill species together with other filter feeders including *Mobula birostris*, *M. kuhlii* and *M. japanica*, and *Balaenoptera edeni* (Authors, unpub. data). These co-occurrences with other megafauna highlight a high degree of prey availability in the area. It is yet unclear if the sharks visit Honda Bay to specifically forage on small fishes or krill, or perhaps both. Their low resighting rate within season, and the low estimated residency as calculated through modified maximum likelihood methods, suggests these whale sharks might visit Honda Bay to opportunistically forage on available prey, before moving elsewhere. Interestingly peaks in productivity as inferred from chl-α are highest between Nov and Feb, like in Lahad Datu, Malaysia, to the south, and like in the Bohol Sea to the east (Authors, unpub. data^54^), and thus it remains unclear the main drivers for these long-distance movements. Unlike other coastal sites where whale sharks are known to spend considerable amounts of time as explained through their lagged identification rate (e.g. ~31 d, Mafia Island, Tanzania^52^ ~27 d, Pintuyan, Southern Leyte^27^; ~50 d, Donsol, Luzon^19^), whale sharks visiting Honda Bay appear to be short-term visitors as that observed in Honduras (~12 d)^53^, the Red Sea (~12 d)^21^ or Mozambique (~5 d)^52^. Further work into their detailed habitat use whilst in Honda Bay will elucidate this, and perhaps molecular approaches to better understand their foraging ecology^52^.

Only five of the ten tags deployed transmitted data. This failure has been reported in pop-up archival tags before with studies reporting ~50% transmission success^55^. This is a considerable rate of failure given the cost of these tags, satellite time, field costs involved, and the invasiveness on the target animals. Individual P-1122 was resighted 103 days after tagging still carrying the tag. It looked heavily fouled and that tag failed to report altogether. It could perhaps be a fouling issue by which the tags become too heavy to pop-up, or even to detach altogether from the animal. Similarly, individual P-1396 was resighted 350 days after tagging (tag never reported) but with no tether and no obvious scar to the naked eye, suggesting the tag, tether and anchor might have been pulled out. Given the cost of these tags and the animal welfare implications, careful considerations should be made given their poor success at least in this region. Nonetheless, results yielding conservation and management implications can arguably outweigh invasive methods employed such as drag from fouled tags or tag deployment. Araujo *et al*.^45^ deployed towed SPOT5 tags on 17 juveniles and obtained basin-wide connectivity data as well as more detailed habitat use data for coastal areas, albeit a shorter tracking time (~64 days). Perhaps an adaptation of these tags (e.g. dorsal fin clamp^56^, or dorsal fin tether^57^) might yield better results.

Time-at-temperature reported herein is similar (66–77%) to that reported elsewhere for juvenile whale sharks in the tropics^38,39,45^, yet warmer than that reported for whale sharks at cooler aggregations^40,55,58^. Interestingly, at localities with higher average water temperatures, size at which 50% of males reach maturity was reportedly smaller (7.0 m, Gulf of Mexico^8^; 7.3 m, Qatar^7^; 6.8 m, Philippines^19^) than that at more temperate localities (8.1 m, Ningaloo Reef^59^; 9.2 m, western Indian Ocean^50^). Perhaps juvenile sharks spending the majority of their time in warmer, steadier conditions, allow for faster growth and development^60^. Further investigation into the growth, temperature and the effects of global ocean warming on this endangered species are necessary.

The mean vertical velocity estimated herein for dives >500 m deep was 0.32 ms^−1^ (max. 0.99 ms^−1^) for 3 sharks of mean 4 m *T*_*L*_. Tyminski *et al*.^61^ reported a mean of 0.68 ms^−1^ in the Gulf of Mexico for 5 sharks ranging from 6–8.5 m *T*_*L*_, with a maximum of 1.83 ms^−1^ descent vertical velocity on a 7.5 m individual. Arguably, these sharks were *ca*. double in size from the ones reported herein which could explain the slower descent velocity. The difference in descent vertical velocity between the two studies could also be a consequence of the sampling rate employed where here we used 7.5–10 min intervals, and Tyminski *et al*.^61^ employed 3 s intervals. This discrepancy in the sensitivity of the data could also explain the slower descent speeds reported here. However, using 5 s intervals at Oslob, Cebu, vertical velocities were slower to those reported herein on similar sized sharks (Authors, unpub. data). It is possible that the speed of descents is dependent on the reason for these deep dives and, for example, a predatory avoidance dive (e.g. leatherback turtle^62^) will likely be at a higher speed than an exploratory dive^63^.

Time-at-depth (97% < 200 m) was similar to that observed in the southern Red Sea (~90% < 200 m^64^), at an offshore aggregation in Qatar (~79% < 50 m^39^), the Seychelles (96% < 100 m^65^), and the Gulf of Mexico (~90% < 200 m^61^). These results are consistent in describing the whale shark as primarily an epipelagic species. However, whale sharks are known to forage deeply and spend considerable amounts of time at deeper waters (e.g. Mozambique^63^; Red Sea^64^; Gulf of Mexico^61^; Arabian Gulf^39^; Philippines^45^). Deep-diving (>200 m) behaviour was observed more frequently during the night (68%) consistent with Araujo *et al*.^45^, Wilson *et al*.^58^ and with Tyminski *et al*.^61^ when the sharks were inshore of the Yucatan Peninsula. Interestingly there were prolonged periods of no deep-diving behaviour at all (Supplementary Fig. 3) that is likely associated with coastal, shallow habitats where there are no adjacent waters >200 m deep, such as Honda Bay. The diel vertical movement is believed to be linked with foraging opportunities^2,58^, in this case, when the sharks are in Honda Bay without access to deep-waters, they stay on the shelf, yet when they leave they likely perform more regular deep dives, and this could explain the periodicity in deep-diving behaviour reported here.

## Conclusions

Our results highlight Honda Bay as a global hotspot for the endangered whale shark where they visit seasonally to feed on small fishes and krill. We used archival tags coupled with photo identification to understand the movements of whale sharks from this area, and report the first international return migration in Asia using these techniques. We demonstrate the usefulness of monitoring social media platforms to generate data on endangered species, and encourage the education of tourists at whale shark hotspots through citizen science programmes to aid monitoring efforts^15^. Coupled with the occurrence of other threatened, endangered and protected (ETP) species, Honda Bay has been declared a Marine Key Biodiversity Area. Understanding critical habitats for whale sharks is one conservation priority for the species^12^, and here we provided evidence that supports Honda Bay as an important habitat, and the strait between Tawi-tawi (PH) and Lahad Datu (MY) as an important migratory corridor for the species.

The whale shark has been protected in the Philippines since 1998 (FAO 193), and in Malaysia since 1999 (Fisheries Regulation of 1999), with a general understanding that poaching is low. However, concerns remain about the illegal take of these animals in the region, in light of fisheries operating in the south of China^13^ that probably have extended fishing grounds into Malay and Filipino waters. Although juvenile whale sharks might not move as much as originally thought^15,52^, they still undergo long-distance movements, or move regionally crossing international boundaries^27,39,40,45,63^ as also reported herein. The results presented here that confirm the movements between the Philippines and Malaysia, and Indonesia, therefore add to this connectivity evidence, and further emphasizes the need for international cooperation to manage this Endangered species. Our results support the objectives of the Coral Triangle Initiative, the Sulu-Sulawesi Seascape Project^66^, and the Concerted Actions for Whale Sharks under CMS (UNEP/CMS/Concerted Action 12.7, 2017) amongst others, to enhance the management and conservation of the whale shark through trilateral collaboration between Indonesia, Malaysia and the Philippines. The whale shark connectivity corridor identified here in the south Sulu Sea including Tawi-tawi and Jolo (PH), Sandakan, Kinabatangan, Kunak and Lahad Datu (MY), appears to be a key area of concern for the species, as has also been highlighted for other ETP species like marine turtles. A trilateral approach will not only raise awareness for the species, contribute essential population monitoring data, identify and mitigate threats, but also act as an umbrella species for other ETP species that require urgent attention (e.g., sharks and rays^67^).

## Methods

### Ethics statement

This study was carried out in accordance with the guidelines and in collaboration with the Department of Agriculture-Bureau of Fisheries and Aquatic Resources, and the Palawan Council for Sustainable Development (PCSD) of the Republic of the Philippines, under whose management the whale shark falls. No animal was restrained during this work. Work was undertaken under PCSD Gratuitous Permit 2017-13 following an initial research proposal wherein the methods employed were detailed.

### Study Site

Honda Bay in Palawan province lies in the northwestern Sulu Sea, a deep-sea habitat (>4,000 m) bounded by Mindoro and Panay Islands to the north, Negros and Mindanao to the east, Borneo and the Sulu Archipelago to the south, and Palawan to the west. The Bay is relatively shallow (<45 m deep), covering 28,000 ha, comprising 12 islands across 19 barangays (villages) within the legislative district of Puerto Princesa City. The Bay hosts over 279 species of fish from 41 families, and 37 genera of hard corals^68^. Honda Bay was highlighted as a Marine Key Biodiversity Area (MKBA) given the occurrence of trigger species, namely globally threatened species within a given habitat of importance. Concerns remain as the fisheries catch declined by almost 10-fold from early 1980s to early 2000s^69^. Whale sharks were reported to occur in numbers by Torres *et al*.^46^, including their direct take. Whale shark tours in Honda Bay have operated since 2009, although no systematic data collection ever took place until this study.

### Boat-based surveys and photo-ID

Dedicated boat-based surveys in Honda Bay were conducted between April 23^rd^ and October 21^st^ 2018. We employed two survey platforms to find whale sharks in the Bay: small outriggered pumpboats (7.9 m, 10 hp), and large tourist bangkas (15 m, 90 hp), similar to those described by Araujo *et al*.^27^. Pumpboat surveys started from Barangay San Jose (9.7982N, 118.7724E), whereas bangka surveys operated from the Puerto Princesa City baywalk (9.7441N, 118.7301E; Fig. 1). Whale sharks were haphazardly searched for within the central and south western part of the Bay (see Fig. 1) when sea state conditions were <Beaufort 3, and swell was <1 m. Birds and fish boils were used as sighting cues to aid in finding the whale sharks. Upon encountering a whale shark, researchers recorded the location on a handheld GPS, entered the water and collected photographic identification of any whale sharks in the water.

### Photo-ID

Photographs of the left and right flank were taken for identification purposes. Left identification images were prioritised over the right, as it’s the current international standard. Only images of sufficient standard that allowed visual identification of the left and right flank were utilised. Whale shark sex was determined by the presence (male) or absence (female) of claspers as confirmed from photographs. No sex was assigned if there was no photographic evidence to confirm it. Maturity in males was externally assessed following Norman and Stevens^59^ and Rohner *et al*.^50^. Maturity in females could not be visually determined. The size of the whale sharks was visually estimated based on items of known length within proximity (i.e. boats and/or other swimmers). Although this method carries an inherent level of error [ref.^70^, it can be used to determine the size class of the study animals^10^. Whale shark behaviour was noted during each encounter and when a whale shark was actively feeding and conditions permitted, a sample of the main prey was collected.

### Data mining

Photo-identification data collected by members of the general public were used to understand whale shark movements. Data was extracted between April and August 2018 from popular social media platforms (i.e. ©Facebook, ©Instagram, ©Twitter and ©YouTube). Systematic searches for specific keywords was conducted using Boolean operators to increase positive matches. Each video or photograph extracted was further processed for identification purposes and inputted into a database including date posted, user, quality and whether it could be used for identification. A second researcher validated extracted and processed images, and visually matched them against a localised database. Where date of encounter could not be verified from the user, the date of upload was used as the encounter date; an assumption necessary to maximise data collection.

### Photo-ID validation

Identification images from dedicated photo-ID effort and different data sources were processed and sorted into separate folders, each corresponding to a different individual whale shark. These were visually matched against a localised database. A second researcher working independently confirmed, sorted and identified images visually. A third researcher, independent from the first two, ran an identification photograph of each individual through the program I^3^S (http://www.reijns.com/i3s)^71^ containing identification images from all sites in the Philippines (Authors, unpub. data). New, unidentified individuals were uploaded onto the online whale shark database ‘Wildbook for Whale Sharks’ (www.whaleshark.org). The presence of each individual whale shark was recorded on a spreadsheet.

### Lagged identification rate

Photo-ID data extracted from data mining sources and collected through dedicated effort was used to calculate the lagged identification rate (LIR), defined as the probability that an individual will be resighted at the study site after a certain time lag^26^. The LIR was modelled using the ‘Movement’ module in the program SOCPROG 2.7^72^, and was used to estimate residency, population size and mortality or permanent emigration. Models were tested for a combination of open, closed, emigration, reimmigration and mortality population parameters (Table 1). This approach operates under the assumption that identified individuals have equal probability of recapture over time, no marks change or can be lost, and individuals can leave and re-enter the site. The quasi-Akaike information criterion (QAIC), was used to account for over-dispersion of the data, and select the best-fitting model^73^. The best-fit model was bootstrapped for 100 repetitions to generate standard errors and 95% confidence intervals^74^.

### Satellite tagging

Ten whale sharks were opportunistically tagged with pop-up archival tags (miniPATs, Wildlife Computers, Washington, USA) between July 19^th^ and 23^rd^ 2017 in Honda Bay, Palawan (Table 2). Upon encountering a whale shark, we used a Hawaiian-sling spear pole to attach the tag to the whale sharks’ left flank, between the base of the dorsal fin and the first lateral ridge. The tag was attached with a titanium dart that is inserted 10 cm into the subdermal tissue of the shark, tethered by a stainless steel line. It is unclear how long darts remain embedded for, but 5 tags recovered by Araujo *et al*.^44^ using the same darts were attached to the tethers upon retrieval. Darts were sharpened and cleaned with 95% ethanol prior to deployment. Tags were deployed on first attempt to minimise disturbance to the animals. Tags were not coated with antifouling. Tags were programmed to pop off after pre-determined intervals of 150, 180 and 300 days (Table 2). Tags recorded on-off-on histograms for depth and temperature for 12 hrs for eight days on, four days off, and four days on, with depth bins of <5 m, 5–15 m, 15–30 m, 30–60 m, 60–100 m, 100–200 m, 200–300 m, 400–500 m, 500–700 m, 700–1,000 m, 1,000–2,000 m, and temperature bins of <0 °C, 0–5 °C, 5–10 °C, 10–15 °C, and then in 2.5 °C increments until 32.5 °C, and then 32.5–45 °C. MiniPATs collected light-level data which was used to determine location. No tags were physically recovered in order to retrieve the full time-series dataset in this study. For those tags that transmitted sufficient depth time-series data, we calculate the number of deep-dives (>200 m) during day and night, and estimate the vertical velocity of extreme dives (>500 m) based on pre-set intervals of 7.5–10 min.

All data retrieved from the tags was transmitted through the ARGOS satellite system and downloaded from Collecte Localisation Satellites (www.argos-system.cls.fr). Wildlife Computers’ DAP3 processor was used to estimate light-level data into geographic locations of the tags deployed. This processor uses a Hidden Markov Model (HMM) with a multi-step algorithm at a 0.25° grid size with light levels, bathymetry data (ETOPO-1)^75^, tagging location (GPS), pop-up location (first ARGOS Location Quality 3), any other GPS location assigned by the user, sea surface temperature (SST; NOAA OI SST V2, http://www.esrl.noaa.gov/psd), and a mean animal speed of 3.5 km h^−1^ ^47,76^. Light-level locations had a mean radius error of ~50 km, and 50%, 95% and 99% confidence intervals were generated (Wildlife Computers, 2018), presented herein as Supplementary Video 1.

## Supplementary information

Supplementary Information

Supplementary Video 1

## Data Availability

All identification data is hosted on the online database ‘Wildbook for Whale Sharks’ (www.whaleshark.org). Tag data will be made freely available upon manuscript publication.
