# Supplementary material for: Photo-ID and telemetry highlight a global whale shark hotspot in Palawan, Philippines
Source: Sci Rep. 2019 Nov 20;9:17209. doi: 10.1038/s41598-019-53718-w (PMC6868279; doi:10.1038/s41598-019-53718-w)
Supplement: Supplementary file 1 — Supplementary Information [file 41598_2019_53718_MOESM1_ESM.docx]

**Supplementary material**

**Photo-ID and telemetry highlight a global whale shark hotspot in Palawan, Philippines**

Gonzalo Araujo^1,*^, Ariana Agustines^1^, Brian Tracey^1^, Sally Snow^1^, Jessica Labaja^1^, Alessandro Ponzo^1^

^1^Large Marine Vertebrates Research Institute Philippines, Cagulada Compound, Jagna, 6308, Bohol

*Corresponding author: g.araujo@lamave.org | +639054043833


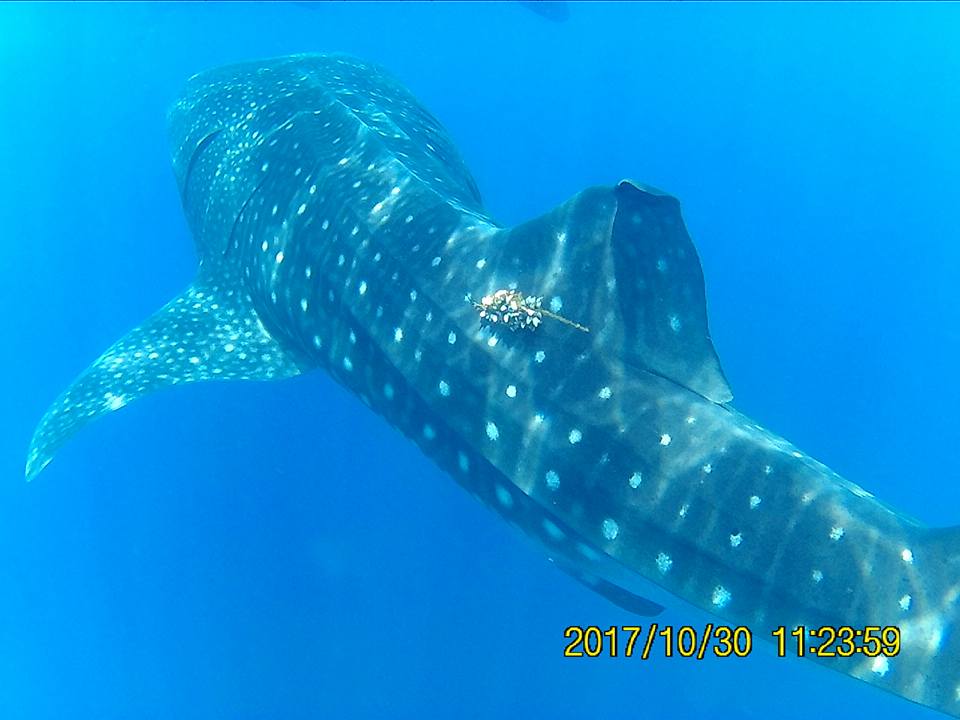


**Supplementary Figure 1.** Whale shark P-1122 resighted on October 30, 2017, carrying a heavily-fouled miniPAT tag in Honda Bay, Palawan. ©Andy Leonor, Dolphin and Whales Travel and Tours.


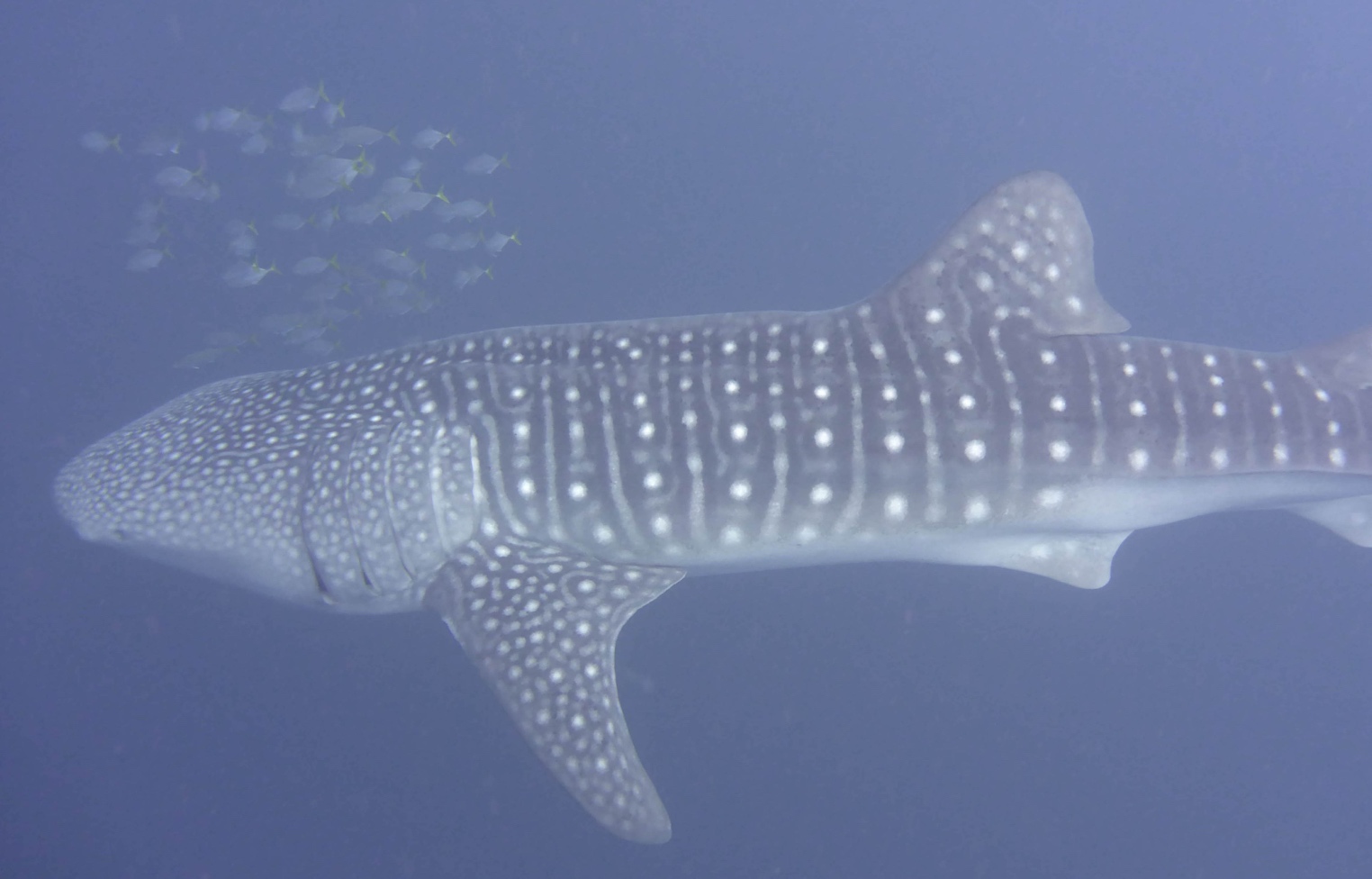


**Supplementary Figure 2.** Whale shark P-1396 resighted ~1 year after initial tagging showing no obvious scars or markings.

**Supplementary Figure 3.** Deep dives (>200 m) by individuals P-1125, P-1126 and P-1128 between July 2017 and January 2018. Note the periodicity in deep-dives over time.

**Supplementary** **Table 1.** Summary of data sourced from online searches on popular social media platforms, obtained directly from the tour operator, a local photographer and from preliminary work by the Authors in 2016 and 2017. Keywords are case sensitive. Only left flank identification images are used to determine the identity of an individual whale shark.

| **Source** | **Keyword used** | **Results** | **Honda Bay IDs Extracted** |
| --- | --- | --- | --- |
| ©Facebook | Butanding Honda Bay | 1 | 1 |
|  | Butanding puerto princesa | 1 | 0 |
|  | Honda Bay Whale Shark | 25 | 9 |
|  | Puerto Princesa whale shark | 4 | 0 |
|  | Whale shark Honda Bay | 1 | 0 |
|  | Whale shark Palawan | 7 | 4 |
|  | Whale Shark Puerto Princesa | 84 | 25 |
| ©Instagram | #hondabay | 27 | 6 |
|  | #palawanwhaleshark | 1 | 0 |
|  | #whalesharkpalawan | 1 | 0 |
|  | whaleman_wanderer | 7 | 1 |
| ©YouTube | Honda Bay Whale Shark | 2 | 0 |
|  | Palawan Whale shark | 2 | 1 |
|  | Puerto Princesa whale shark | 16 | 8 |
| Dolphin and Whales Travel and Tours | - | 24 | 24 |
| Duncan Murrell | - | 27 | 27 |
| Authors (2016) | - | - | 10 |
| Authors (2017) | - | - | 10 |
|  | **Total** | **230** | **126** |
|  |  | **Individual IDs** | **86** |

**Supplementary Video 1.** Most probable track for the five tags that transmitted data, with confidence intervals in red (50%), dark orange (95%) and light orange (99%).
